# Screening of CRISPR/Cas9 gRNA for mimicking Powdery Mildew resistant MLO ol-2 mutant

**DOI:** 10.6026/97320630017637

**Published:** 2021-06-30

**Authors:** Archana Prajapati, Vikrant Nain

**Affiliations:** 1 School of Biotechnology, Gautam Buddha University, Greater Noida 201312, India

**Keywords:** Genome editing, Powdery mildew resistance, CRISPR/Cas9, SlMLO1 locus, Oidium neolycopersici

## Abstract

Powdery Mildew (PM) caused by fungal pathogen Oidium neolycopersici (O. neolycopersici) affects both greenhouse and field-grown tomato production. Resistance to PM disease can be achieved by selective inactivation of Mildew Resistance Locus O (MLO) genes encoding heptahelical transmembrane domains, which confer susceptibility to fungal pathogens. Natural loss-of-function mutation is a 19 base pair (bp) deletion in the SlMLO1 gene locus responsible for fungal resistance in S. lycopersicum var. cerasiforme. Introgression of these resistance alleles through breeding into elite varieties is possible. However, this is a long and labour-intensive process and has limitations due to linkage drag. Nonetheless, recent developments in the field of genome editing technology particularly CRISPR/Cas9 systems allows quick, effective and accurate genome modification at the target gene locus. Therefore, it is of interest to determine the efficacy and exact deletion that mimics the natural ol-2 (Slmlo1) mutation present in wild tomatoes using CRISPR/Cas9. 947 putative guide RNAs (gRNAs) were designed using Cas9 variants to broaden Protospacer Adjacent Motif (PAM) compatibility and to enhance DNA specificity against the SlMLO1 locus. 60 out of 947 gRNAs were selected based on the recognition of the PAM sequence, the MIT specificity ranking, the off-target sites, their distance from the 19bp natural ol-2 mutation, the secondary structure of the gRNAs, and their minimum free energy. In depth analysis of these 60 gRNAs helped in the selection of the top five gRNAs based on the above-mentioned criteria. These gRNAs are useful for introducing deletions identical to natural ol-2 mutants and impart resistance against fungal pathogen O. neolycopersici in cultivated tomato crops.

## Background

Food demand will continue to rise with the growing global population, at least until 2050, when the global population is projected to stabilize. There will be an estimated 9.7 billion people in 2050, and we will need about 70 percent more food available for human consumption than is consumed today [1]. Therefore, the food and nutrition security need to be addressed by developing crop varieties that are disease-resistant, stress-tolerant, high yielding and have improved nutritional benefits [1]. Tomato is the second most valuable fruit or vegetable crop next to potato, with approximately 182.3 million tonnes of tomato fruit grown each year on 4.85 million hectares worldwide [2]. Asia accounts for 61.1 percent of global tomato production, while Europe, America, and Africa produce 13.5 percent, 13.4 percent, and 11.8 percent of total tomato yield, respectively [2]. However, tomato production is limited by several abiotic and biotic constraints [2]. Powdery Mildew (PM) is a widespread fungal disease affecting thousands of plant species, caused by ascomycete fungi belonging to the order of Erysiphales [3, 4]. The disease is macroscopically characterized by ‘powdery’ fungal reproductive structures on the surface of plant organs especially leaves and it causes significant harvest loss in crop plants such as tomato, wheat, and barley [3]. Powdery Mildew caused by Oidium neolycopersici (O. neolycopersici) has been recognized as a worldwide emerging pathogen on tomato [5]. Chemical control of the PM is possible by fungicides but this increases farming costs and public concerns related to environmental pollution and human health [4]. Therefore, the development of the tomato cultivars carrying resistance genes is the best approach to control the PM disease. Specific homologs of the plant Mildew Locus O (MLO) gene family act as susceptibility factors towards the PM disease causing significant economic losses in agricultural settings [4]. MILDEW RESISTANCE LOCUS O (MLO) wild-type alleles encode a membrane-associated protein with seven transmembrane domains (TM), topologically reminiscent of metazoan G-protein coupled receptors and are conserved in monocots and dicots, confer susceptibility to fungi that cause the PM disease [4], [6]. Powdery mildew resistant mlo mutants were first described in Hordeum vulgare (barley) and later characterized in model plant Arabidopsis thaliana. Since then the following plant species have been identified in chronological order for mlo-based resistance: tomato (Solanum lycopersicum), pea (Pisum sativum), strawberry (Fragaria vesca),pepper (Capsicum annuum), bread wheat (Triticum aestivum), cucumber (Cucumis sativus), rose (Rosa hybrida), tobacco (Nicotiana tabacum), melon (Cucumis melo), grapevine (Vitis vinifera), and apple (Malus domestica) [7]. The ol-2 allele originates from a wild accession of S. lycopersicum var. cerasiforme (cherry tomato), a close relative of common cultivated tomato mediating recessively inherited broadspectrum resistance to the PM pathogen O. neolycopersici [5,6]. The histological analysis of ol-2 reveals the early abortion of fungal pathogenesis at the site of infection due to the formation of plant cell-wall appositions or papilla formation [5, [6]. Also, at the plantpathogen interaction sites, PM-based resistance by mlo is associated with the enhancement of exocytosis defense pathways, which are thought to contribute to the prevention of fungal penetration into host cells [4]. Experimental data indicate that the tolerance of mlo is not unique to specific fungal isolates and is highly durable [4]. There are 16 MLO genes in tomatoes, SlMLO1 to SlMLO16 and SlMLO1 is the primary contributor towards susceptibility to powdery mildew and homozygous loss-of-function mutations (Slmlo1) result in resistance to powdery mildew [8]. Since these natural loss-of-function slmlo1 mutants are present in tomatoes,these can be introgressed in the susceptible varieties through classical breeding. However, introducing these alleles into elite varieties through breeding is a long and labor intensive process and has limitations due to linkage drag. This process is accelerated using efficient genome editing techniques. Emergence of genome editing techniques Zinc-finger nucleases (ZFNs), transcriptional activator-like effector nucleases (TALENs), Clustered Regularly interspaced short Palindromic Repeats/ Associated protein-9 nuclease (CRISPR/Cas9) system have made it possible to introduce desired modification at the selected locus in diverse species revolutionizing plant breeding [9]. CRISPR/Cas9 systems are adaptive immune systems that confer resistance against phages and invading nucleic acids in archaea and bacteria [10]. CRISPR/Cas9 system is a type II system derived from Streptococcus pyogenes consisting of mature CRISPR RNA (crRNA) and trans-activating crRNA (tracrRNA), the gRNA (tracrRNA with crRNA) guides the Cas9 endonuclease to bind and then cleave the specific target sequence [10,11]. Cas9 has a bilobed gRNA capture structure with alpha-helical and nuclease lobes, composed of HNH and RuvC domains [12]. Cas9 endonuclease uses its HNH nuclease domain to cleave the target DNA strand complementary to gRNA and cleave the DNA strand non-complementary to sgRNA by its RuvC like nuclease domain, producing a blunt end double-strand break (DSB), about 3-4 nucleotide upstream of protospacer adjacent motif (PAM) sequence [13]. DSBs at the target site could be repaired by error-prone non-homologous end joining (NHEJ) or donordependent homologous recombination (HR) pathway [9].

Amongst ZFNs, TALENs, and CRISPR/Cas9 genome editing systems, CRISPR/Cas9 are comparatively easy to design, costeffective, efficient than TALENs, and ZFNs [14]. CRISPR/Cas9 is used in many crop plant species consisting of rice, wheat, maize and tomato [14]. Proof of concept for this strategy has been provided by the TALENbased approach to successfully introduce PM resistance in bread wheat through simultaneous targeting of three homeolog of MLO alleles [4]. Also, a recent study reported CRISPR/Cas9 mediated knockdown of SlMLO1 in tomatoes to achieve resistance against the PM. Tomelo is a non-transgenic plant variety resistant to powdery mildew fungal pathogen O. neolycopersici produced using CRISPR/Cas9 technology without any off-target mutation [15]. ol-2 mediated PM resistance against O. neolycopersici has been observed in natural accession LA-1230, Solanum lycopersicum var. cerasiforme [5]. Therefore, it is of interest to determine the efficacy and exact deletion that mimics the natural ol-2 (Slmlo1) mutation present in wild tomatoes using CRISPR/Cas9.

## Methodology

### Gene Sequences

The complete genomic, cDNA, and protein sequence of the SlMLO1 gene (GenBank Accession AY967408.1) was retrieved from Sol Genomics Network [16,17]. Physicochemical properties of proteins viz. Isoelectric point (pI) and molecular weight were calculated using the Expasy tool [18]. Multiple sequence alignments were carried out using BioEdit [19]. InterProScan database was used to determine the transmembrane, cytoplasmic and non-cytoplasmic domains of the SlMLO1 wild type and natural ol-2 protein sequence [20,21].

### Homology Modelling of SlMLO1 protein

The structural model of the SlMLO1 protein was predicted using the I-TASSER web server and visualized with ViewerLite [22]. Structural alignment of native and truncated Slmlo1 proteins was done using the SuperPose web server [23].

### gRNA designing and evaluation:

CRISPOR online tool was used for the identification of potential gRNA and potential off-target sites [24]. gRNAs were designed for 20 known Cas9 enzyme variant options [25]. gRNA secondary structures and free energies were calculated using the RNA fold web server using default parameters [26,27].

## Results and Discussion:

**Figure 1:**
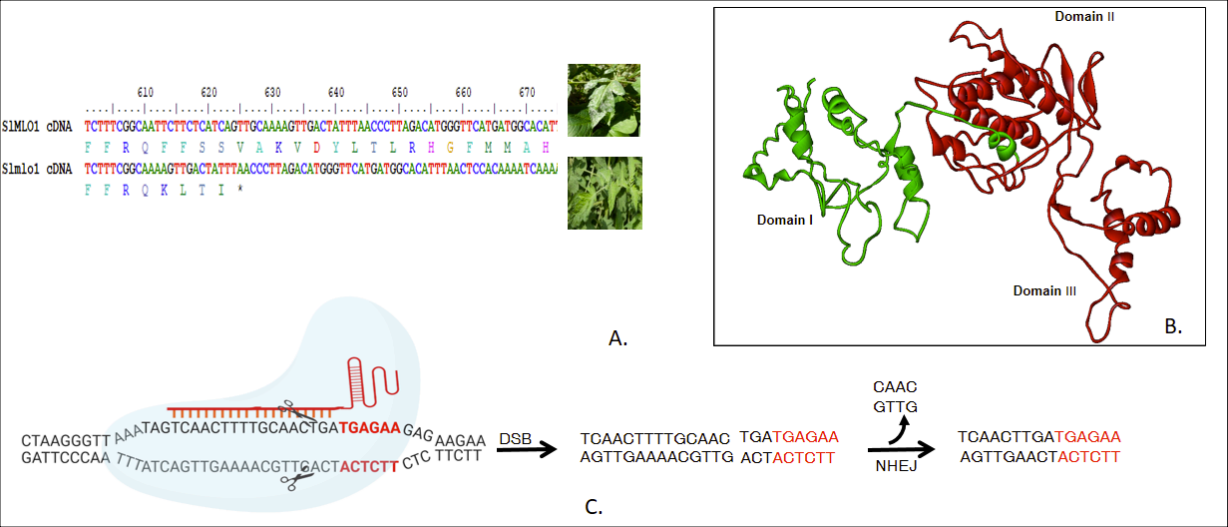
Comparison of native and ol-2 SlMLO1 sequences. A. cDNAsequence alignment of the SlMLO1 and Slmlo1 (ol-2) mutant highlights the region of 19 bpnatural deletion. B. Superposed image of SlMLO1 and Slmlo1 protein using SuperPosev1.0 web server. The image indicates the absence of domains II and III in ol-2 mutants. C. Schematic representation of CRISPR/Cas9 based introduction of lossof-function mutation of SlMLO1 locus mimicking the natural ol-2 mutation. The nuclease domains of Cas9 cleave three base pairs upstream of PAM creating Double-strand breaks (DSBs), further repaired by Non-homologous End joining (NHEJ).

**Figure 2:**
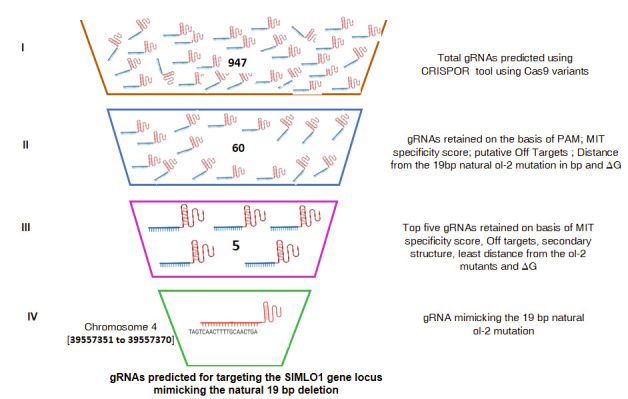
Screening of gRNAs against SlMLO1 locus. (i) Total gRNAs predicted using CRISPOR tool for Cas9 variants againstSlMLO1locus;(ii–iv) Screening of predicted gRNAs on the basis of PAM; MIT specificity score; putative Off-targets; Distance from the 19bp natural ol-2 mutation in bp and 'G, espectively. Roman numerals (at the left side of the figure) indicate stages of screening of gRNAs. Numbers inside the figure indicates the number of gRNAs at different stages of screening. Details provided for gRNA at the last stage of screening represents gRNA mimicking the exact19 bp natural ol-2 mutations in the target SlMLO1 locus.

The present study aims at designing and screening of CRISPR/Cas9 gRNA against the target SlMLO1 gene locus that mimics the natural ol-2 mutation present in wild tomato, resistant against PM pathogen O. neolycopersici. This CRISPR/Cas9 mediated targeted genome modification will have a very high potential to introduce fast, efficient, and specific mutation in cultivated tomato crops, leading to the incorporation of PM resistance. The SlMLO1 gene (5.5 kb) consists of 15 exons and 14 introns, coding for 1.5 kb mRNA and 58.36 kDa polypeptide of isoelectric point 8.93. Multiple Sequence alignment of wild type and mutant SlMLO1 cDNA showed a 19 bp deletion (39557361..39557379, GenBank Accession number: CP023760.1) at Chromosome 4 in 7th Exon of the ol-2, that is responsible for resistance in S. lycopersicum var. cerasiforme in cultivated tomato Solanum lycopersicum (Figure 1A). The 19 bp deletion leads to frameshift mutation resulting in a truncated nonfunctional Slmlo1 protein. InterProScan results show the existence of possible seven hydrophobic TM domains and four cytoplasmic domains in SlMLO1 protein. On the other hand, Slmlo1 has three TM domains and 19 bp mutations located as part of the second cytoplasmic domain and suggested for the truncated second cytoplasmic domain. This indicates that the remaining four transmembrane domains may be involved in conferring disease susceptibility. The length of the expressed Mlo protein was shortened by 60.15% by the frameshift mutation. A research study showing the comparable observation accidentally observed a point nucleotide mutation during cloning causing the replacement of retained glutamine residue with arginine (Q198R) in the NtMLO1 protein sequence leading to complete gene loss of function [4]. Homology based 3D structural model of SlMLO1 protein shows that it has three functional domains. Further structural superposition of both native and ol-2 mutant protein shows that ol- 2 mutant lacks domains II and III (Figure 1B). This indicates the possible biological role of domain I in plant physiology or protection and domains II and III may be required for PM pathogenesis in susceptible varieties. Previous studies have reported that the PM susceptibility-mediating activity of the MLO protein is due to or enhanced by calcium-dependent calmodulin (CaM) binding to the Calmodulin binding domain (CaMBD) in the cytoplasmic carboxy-terminal protein region [3]. A total of 947 potential gRNAs were identified using CRISPOR, for 20 different Cas9 variants (Figure 2). Further, the top 60 gRNAs representing three top-ranking gRNA for each Cas9 variant were selected for further evaluation.

**Figure 3:**
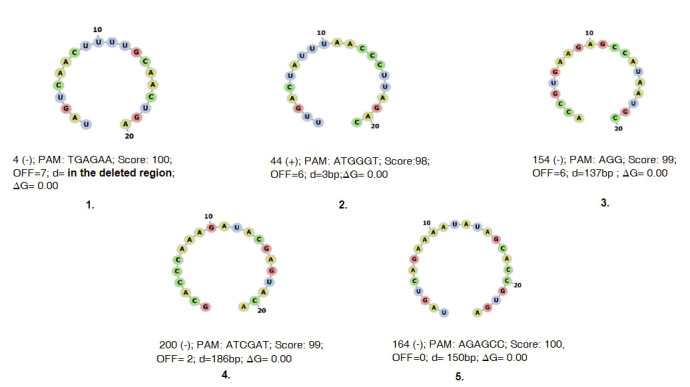
Top five potential gRNAsselected out of nine hundred forty-seven potential gRNAson the basis of PAM Motif (PAM); MIT specificity score (Score); potential number off-targets (OFF); distance from the 19bp natural ol-2 mutation (d) and minimum free energy ('G). First guide RNA was selected as it lies exactly in the region of 19 bp natural ol-2 mutation in the SlMLO1 gene locus.

**Figure 4:**
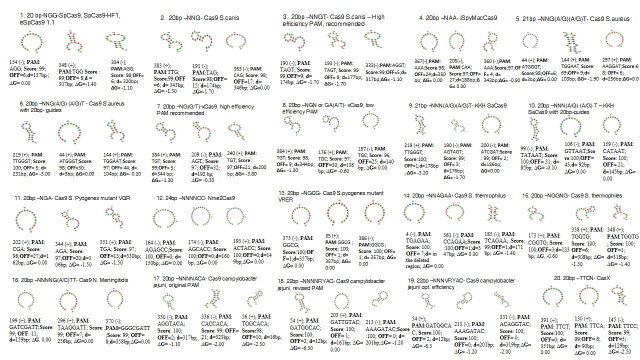
Top sixty potential gRNAs selected out of nine hundred forty-seven potential gRNAs on the basis of PAM Motif (PAM); MIT specificity score (Score); potential number off targets (OFF); distance from the 19bp natural ol-2 mutation (d) and minimum free energy (ΔG)

The twenty Cas9 variants were selected to determine the specificity of gRNAs that could recognize different PAM sequences other than NGG (SpCas9). The top 60 gRNAs were selected based on the recognition of the PAM sequence, the MIT specificity ranking, low off-target similarity, their distance from the 19 bp natural ol-2 mutation, the secondary structure of the gRNAs, and their minimum free energy (Figure 4). Out of these 60 putative gRNAs, the top five gRNAs were further selected to improve the precision of editing with minimal or no off-target effects on the basis of MIT specificity ranking, least distance from the 19 bp natural ol-2 mutation, secondary structure, and minimum free energy requirement. The top five gRNAs did not show any secondary structure and they all have zero free energy, indicating that these gRNAs are more accessible to binding to target gene locus and thus increase the efficiency of editing (Figure 3). Out of these five gRNAs, we found one gRNA targeting the region similar to the 19 bp deletions of ol-2 mutants.

## Conclusion

gRNA mimicking the ol-2 mutants was designed against the SlMLO1 gene locus for conferring resistance against the PM. Moreover, screening of these gRNAs on the basis of PAM sequence,the MIT specificity ranking, low off-target similarity, their distance from the 19 bp natural ol-2 mutation, the secondary structure of the gRNAs, and their minimum free energy increases the specificity and efficiency of gene editing to SlMLO1 gene locus. These designed gRNAs are expressed under a constitutive promoter for inducing PM resistance in cultivated tomato crop as wild type PM resistant tomato.
